# Transcriptome-Wide Identification and Expression Profiling Analysis of Chrysanthemum Trihelix Transcription Factors

**DOI:** 10.3390/ijms17020198

**Published:** 2016-02-02

**Authors:** Aiping Song, Dan Wu, Qingqing Fan, Chang Tian, Sumei Chen, Zhiyong Guan, Jingjing Xin, Kunkun Zhao, Fadi Chen

**Affiliations:** College of Horticulture, Nanjing Agricultural University, Nanjing 210095, China; aiping_song@aliyun.com (A.S.); 2013104120@njau.edu.cn (D.W.); 2013104100@njau.edu.cn (Q.F.); 2015204031@njau.edu.cn (C.T.); chensm@njau.edu.cn (S.C.); guanzhy@njau.edu.cn (Z.G.); 2014104098@njau.edu.cn (J.X.); 2014104099@njau.edu.cn (K.Z.)

**Keywords:** *Chrysanthemum morifolium*, phylogenetic analysis, stress response, transcription pattern, trihelix

## Abstract

Trihelix transcription factors are thought to feature a typical DNA-binding trihelix (helix-loop-helix-loop-helix) domain that binds specifically to the GT motif, a light-responsive DNA element. Members of the trihelix family are known to function in a number of processes in plants. Here, we characterize 20 trihelix family genes in the important ornamental plant chrysanthemum (*Chrysanthemum morifolium*). Based on transcriptomic data, 20 distinct sequences distributed across four of five groups revealed by a phylogenetic tree were isolated and amplified. The phylogenetic analysis also identified four pairs of orthologous proteins shared by *Arabidopsis* and chrysanthemum and five pairs of paralogous proteins in chrysanthemum. Conserved motifs in the trihelix proteins shared by *Arabidopsis* and chrysanthemum were analyzed using MEME, and further bioinformatic analysis revealed that 16 *CmTHs* can be targeted by 20 miRNA families and that miR414 can target 9 *CmTHs*. qPCR results displayed that most chrysanthemum trihelix genes were highly expressed in inflorescences, while 20 *CmTH* genes were in response to phytohormone treatments and abiotic stresses. This work improves our understanding of the various functions of trihelix gene family members in response to hormonal stimuli and stress.

## 1. Introduction

Plant growth and productivity are under constant threat from environmental changes in the form of biotic and abiotic stresses. With a very important role in signal transduction in plants in response to stress [1], transcription factors (TFs) bind to specific gene promoter regions and *cis*-acting elements to activate or inhibit transcription [2].

There are over 60 TF families in plants [3], and their functions are gradually being defined. A growing number of TFs belonging to such families as *MYB* [4] and *WRKY* [5] have been linked to responses to environmental stress in chrysanthemum. In contrast, studies on trihelix TFs are limited to date, though this family has recently attracted attention. This family (also known as GT factors) was named according to its conserved DNA-binding domain, which contains three tandem helices (helix-loop-helix-loop-helix) that bind specifically to the GT motif, a light-responsive DNA element. The DNA-binding domain of GT factors are rich in basic and acidic amino acids, as well as proline and glutamine residues, and GT elements are highly degenerate cis-elements with A/T-rich core sequences [6]. The amino acid sequences of these functional domains exhibit a high degree of conservation, also typically with similarity at either the N-terminus or C-terminus.

The trihelix family has been divided into GT-1, GT-2, GTγ, SH4, and SIP1 5 subfamilies [3], with the name of each clade based on the first member identified. GT-1 clade proteins (GT-1 and GT-3) with one trihelix DNA-binding domain specially bind to Box II (5′-GTGTGGTTAATATG-3′) and the 5′-GTTAC-3′ motif, respectively [7]. GT-2 types have two DNA TF domains: a C-terminal domain that recognizes the GT-2 box (5′-GCGGTAATTAA-3′) and an N-terminal domain that recognizes the GT-3 box (5′-GAGGTAAATCCGCGA-3′) [8]. The C-terminus of trihelix family members show large differences in structure, often forming a long α-helix domain that is predicted to form a coiled-coil structure [9]. Homology analysis reveals higher similarity between the GT-1 and GT-2 clade compared to the others.

Although knowledge of this family was confined to regulation of light-responsive genes [10], recent studies have indicated that the trihelix family also has important functions in different processes of growth and development involving flowers, stomata, trichomes, embryos, and seeds. Trihelix family genes not only participate in disease resistance and salt tolerance, but also in response to cold and drought [11]. In soybean, 63 *GT* genes were found to respond to stress, with at least 11 involved in responses to biotic or abiotic stress. *PETAL LOSS* (*PTL*), belonging to the GT-2 clade, was the first trihelix gene identified as being associated with floral organ morphogenesis [12]. *GmGT2B* acts both in plant stress tolerance but also reduces sensitivity to abscisic acid (ABA) [13]. Belonging to the GT-2 clade, the *GT-2-LIKE1* (*GTL1*) gene has been identified as a trihelix TF related to trichomes, with increased levels of polyploidy and reduced stomatal numbers occurring with mutations [14]. *PtaGTL1* of poplar demonstrates high homology to *Arabidopsis* *AtGTL1* and is also involved in the development of stomata and trichomes [15]. Genetic analyses revealed that the expression of *SHAT1* in abscission zone was positively regulated by the trihelix transcription factor *SH4*, which is involved in regulating traits of rice grain separation [16]. A gene of the SIP1 family (At3g10030) was reported to be associated with leaf development, as the corresponding mutant displayed a short stature, leaf distortion, and a light green color [17].

Chrysanthemum (*Chrysanthemum morifolium* Ramat.) is a popular ornamental species that is globally considered second to rose in its market value [18]. With the rapid development of molecular biology, the molecular genetic improvement of chrysanthemum is increasingly becoming a hot topic. As trihelix TFs interact with cis-elements present in the promoter regions of several stress-related genes, regulating their expression to enhance plant stress tolerance, they may play a critical role in stress signaling transduction pathways. Trihelix TFs genes have been cloned in a variety of plants but mainly model plants, such as 30 members in *Arabidopsis thaliana* and 31 in rice. Conversely, there are few reports related to chrysanthemum. Here, we report the isolation of 20 chrysanthemum trihelix TFs based on a set of transcriptomic data. We performed a comparative phylogenetic analysis of the chrysanthemum genes with those in *Arabidopsis*. Some plant hormones, such as ABA, salicylic acid (SA), and methyl jasmonate (MeJA), are involved in the response to various stresses by activating the transcription of several defense-related genes. For example, SA and MeJA coordinately function in biotic stress signaling upon pathogen infection [19], and ABA is extensively involved in the response to various biotic and abiotic stresses, including pathogen infection, cold, and drought stress [20]. In order to speculate function of 20 chrysanthemum trihelix TFs, we further analyzed the effect of various stress and phytohormone treatments on transcription levels.

## 2. Results

### 2.1. Identification and Phylogenetic Analysis of Putative Trihelix Factors in Chrysanthemum

Twenty chrysanthemum trihelix gene sequences were isolated and designated *CmTH1* through *CmTH20* (GenBank: KT253111-KT253130). The full-length cDNAs varied in length from 724 to 2421 bp, with predicted protein products from 211 (CmTH18) to 684 (CmTH16) amino acids. Details regarding the *CmTH* sequences are given in Table 1. Twelve CmTH proteins are predicted to be nuclearly localized, excepting CmTH4, 6, 7, 9, 10, 11, 12, and 18, in which a conserved bipartite nuclear localization signal (NLS) was not found. The predicted localizations are provided in Table 1.

**Table 1:** Summary of *CmTH* sequences and the identity of likely *A. thaliana* homologs AdMyb/SANT = alcohol dehydrogenase transcription factor Myb/SANT-like family protein HLP = homeodomain-like superfamily protein SSDB-TF = sequence-specific DNA binding transcription factors DHLP = duplicated homeodomain-like superfamily protein. pI, isoelectric point; *M*w, molecular weight.

Gene	GenBank Accession No.	Amino Acids Length (aa)	AtTH Orthologs	Locus Name	pI	*M*_W_	Subcellular Loclization
*CmTH1*	KT253111	340	*AdMyb/SANT*	AT2G44730.1	9.51	38,618.98	nucleus
*CmTH2*	KT253112	314	*AdMyb/SANT*	AT2G44730.1	9.09	35,364.01	nucleus
*CmTH3*	KT253113	338	*AdMyb/SANT*	AT2G44730.1	6.13	38,292.92	nucleus
*CmTH4*	KT253114	224	*AdMyb/SANT*	AT2G44730.1	9.56	24,572.87	chloroplast stroma
*CmTH5*	KT253115	295	*AdMyb/SANT*	AT2G44730.1	8.52	33,510.27	nucleus
*CmTH6*	KT253116	422	*ASIL2*	AT3G14180.1	9.27	47,237.56	chloroplast thylakoid space
*CmTH7*	KT253117	399	*HLP*	AT3G25990.1	6.85	45,568.92	microbody
*CmTH8*	KT253118	249	*SSDB-TF*	AT3G54390.1	9.30	27,119.85	nucleus
*CmTH9*	KT253119	446	*SSDB-TF*	AT1G21200.1	6.35	51,562.20	cytoplasm
*CmTH10*	KT253120	384	*SSDB-TF*	AT1G21200.1	6.26	43,610.86	cytoplasm
*CmTH11*	KT253121	357	*AdMyb/SANT*	AT3G24490.1	4.53	41,753.36	mitochondrial matrix space
*CmTH12*	KT253122	216	*SSDB-TF*	AT5G05550.2	9.18	24,860.42	cytoplasm
*CmTH13*	KT253123	373	*AdMyb/SANT*	AT3G24490.1	5.04	42,980.38	nucleus
*CmTH14*	KT253124	372	*AdMyb/SANT*	AT3G24490.1	5.00	42,911.32	nucleus
*CmTH15*	KT253125	382	*AdMyb/SANT*	AT3G24490.1	4.89	43,488.77	nucleus
*CmTH16*	KT253126	684	*DHLP*	AT1G76880.1	6.68	76,768.06	nucleus
*CmTH17*	KT253127	597	*DHLP*	AT1G76880.1	5.31	66,479.62	nucleus
*CmTH18*	KT253128	211	*SSDB-TF*	AT5G05550.2	5.1	23,338.93	cytoplasm
*CmTH19*	KT253129	341	*HLP*	AT5G47660.1	9.62	39,351.70	nucleus
*CmTH20*	KT253130	526	*DHLP*	AT5G28300.1	5.75	61,139.47	nucleus

**Figure 1:**
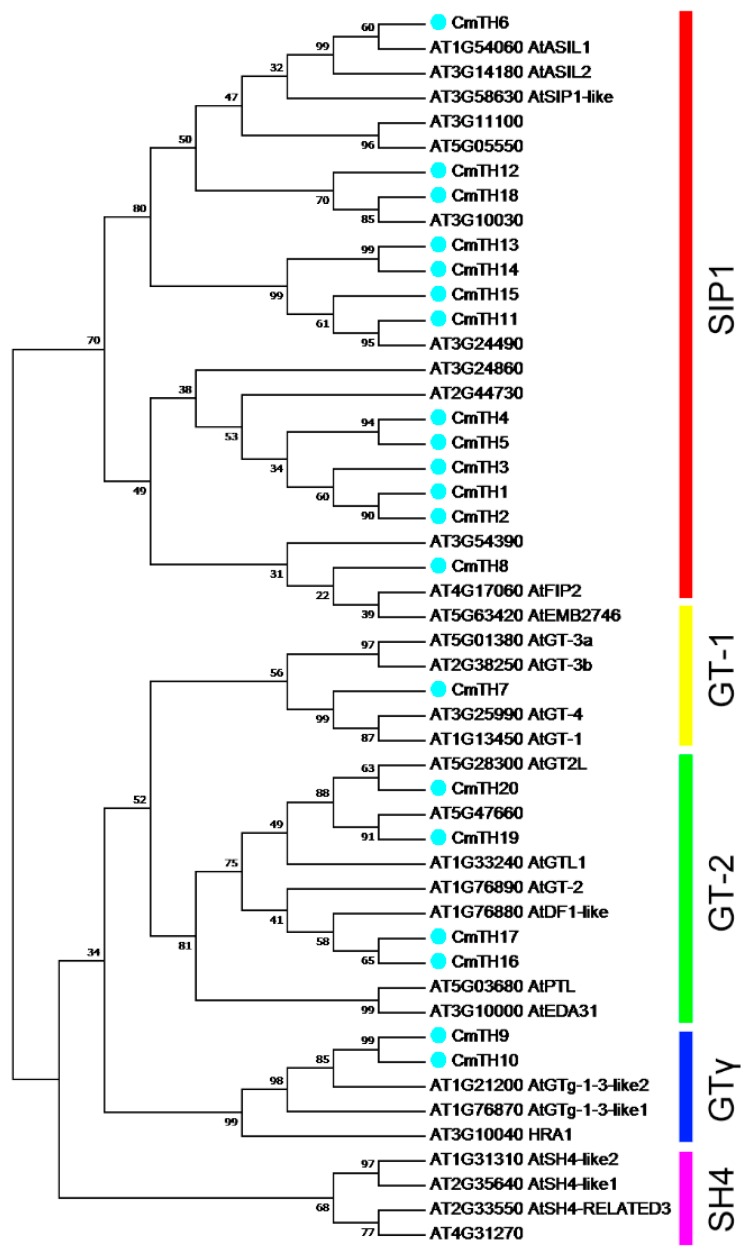
Phylogenetic tree and distribution of conserved motifs among *Arabidopsis* and chrysanthemum TH proteins. The tree was constructed based on a complete alignment of 30 *Arabidopsis* and 20 chrysanthemum trihelix proteins using the neighbor-joining method.

To evaluate evolutionary relationships between *Arabidopsis* and chrysanthemum TH proteins, the deduced amino acid sequences of the TH genes identified were completely aligned. A combined phylogenetic tree (Figure 1) was then constructed using the neighbor-joining method and bootstrap analysis (1000 reiterations), showing diversification among the plant GT family (Figure 1). The twenty *CmTH* genes were found to be distributed across four of five groups, with the exception of the SH4 clade. Furthermore, four pairs of orthologous proteins were identified in *Arabidopsis* and chrysanthemum, AT3G10030 with CmTH18, AT3G24490 with CmTH11, AT5G47660 with CmTH19, AT5G28300 (AtGT2L) with CmTH20, and five pairs of paralogous trihelix family proteins were identified in chrysanthemum: CmTH1 with CmTH2, CmTH4 with CmTH5, CmTH9 with CmTH10, CmTH13 with CmTH14, and CmTH16 with CmTH17.

### 2.2. Conserved Sequences in TH Proteins

**Figure 2:**
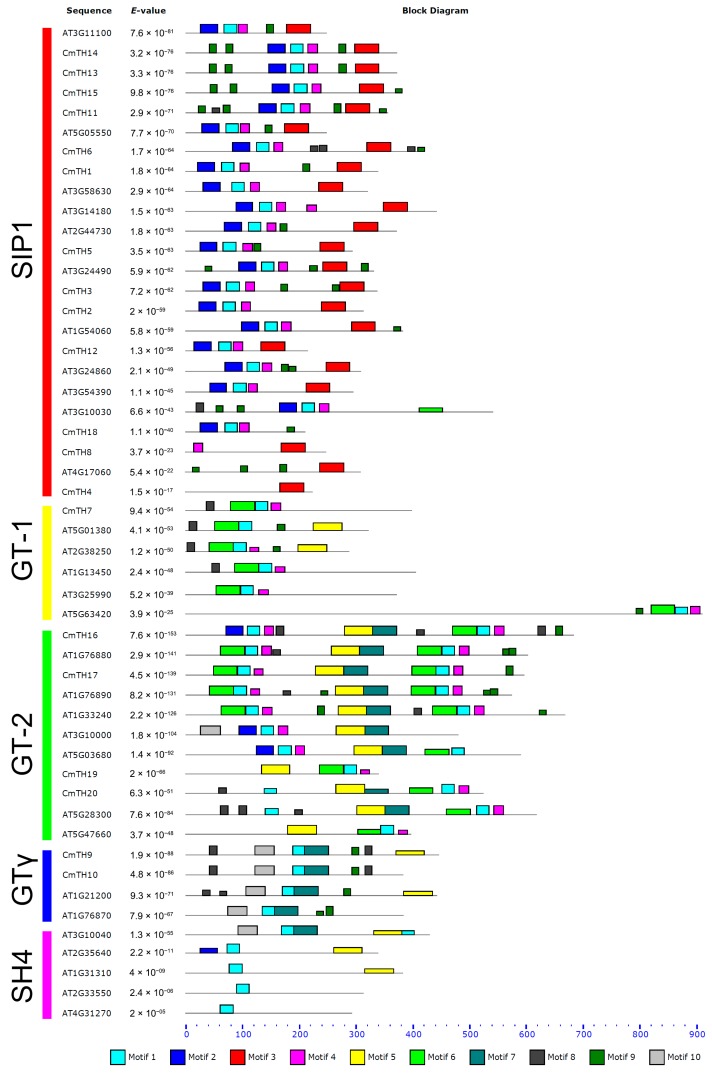
The trihelix protein motifs as derived by MEME analysis. The sequences of motifs of trihelix proteins shown in Figure S1.

The MEME software was used to predict the motif composition of the trihelix factors, and 10 putative motifs with E values less than 1.8 × 10^−45^ were identified (Figure 2 and Figure S1). The trihelix TFs of chrysanthemum can be clearly classified into five subgroups based on the composition of motifs (Figure 2). For example, Motif 2 is mainly shared among the SIP1 clade; Motif 3 is only present in the SIP1 clade, whereas Motif 5 is only absent from this clade. Motif 4 is not found in the SH4 or GTγ clade, and Motif 10 is mainly shared among GTγ clade members. The GT-1 and GT-2 subfamilies possess Motif 6, whereas the GT-2 and GTγ subfamilies contain Motif 7 (Figure 2). Details on these motif features are shown in Figure S1.

The conserved sequences of these motifs were searched against the InterPro database. Motif 1 matched a Myb-type HTH (helix-turn-helix) DNA-binding domain (IPR017930), though no significant matches were retrieved for the other motifs. Most of the CmTH proteins (except for CmTH4 and CmTH8) contain a Myb-type DNA-binding domain (Motif 1), that is usually located near the N-terminus.

### 2.3. miRNA Target Site Prediction

All available plant miRNA data were used to predict candidates targeting *CmTH* transcripts. As shown in Table S1, 16 *CmTH*s are predicted to be targeted by 20 miRNA families, with inhibition of cleavage and/or translation. *CmTH14* and *CmTH15* contain three target sites and *CmTH11*, *13* and *17* two target sites; the other 11 *CmTH*s have only one target site. Additionally, miR414 can target nine of the *CmTH*s.

### 2.4. Transcription Profiling of CmTH Genes

**Figure 3:**
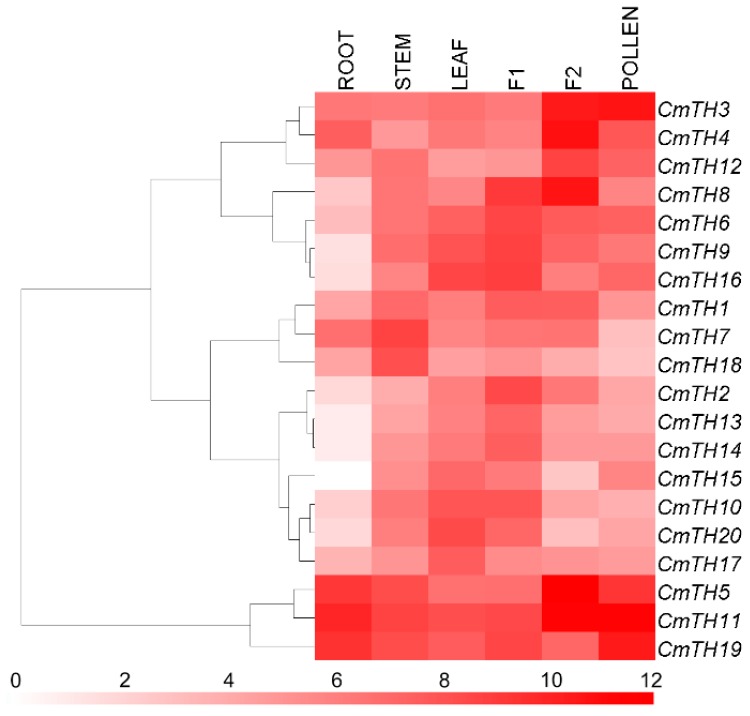
Differential transcription of *CmTH* genes. F1: tubular florets, F2: ray florets at the budding stage. White and red indicate lower and higher transcript abundance, respectively. Bar at the bottom represents log_2_ transformed values.

Since no trihelix factors in chrysanthemum have been previously documented, we investigated the expression profiles of these genes. The results showed differential expression of the 20 *CmTH* genes throughout the plant (Figure 3). However, the expression of *CmTH11* in ray florets was more than three orders of magnitude higher than that of *CmTH15* in roots. Interestingly, the *CmTH13* and *CmTH14* paralogs exhibited a similar expression pattern, whereas the other four pairs of paralogs showed different patterns.

### 2.5. Expression of *CmTH* Genes after Treatment with Phytohormones

**Figure 4:**
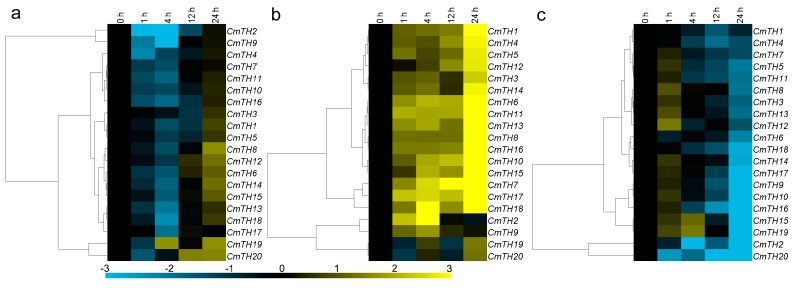
Differential transcription of *CmTH* genes in leaves as induced by the exogenous supply of (**a**) abscisic acid (ABA); (**b**) methyl jasmonate (MeJA); and (**c**) salicylic acid (SA) treatments. Blue and yellow indicate lower and higher transcript abundance, respectively, compared to the relevant controls. Bar at the bottom represents log_2_ transformed values.

Eleven of the twenty *CmTH* genes were significantly down-regulated by exogenous ABA, though *CmTH6*, *CmTH8*, *CmTH12*, *CmTH14*, and *CmTH15* were induced at 24 h. In contrast, *CmTH19* and *CmTH20* transcripts were increased at 4/24 and 12/24 h after ABA treatment, respectively, whereas the expression of *CmTH1*, *CmTH3*, *CmTH5*, *CmTH7*, *CmTH10*, *CmTH11*, and *CmTH15* was not affected by ABA (Figure 4a). The chrysanthemum *TH* family genes displayed three main expression patterns under MeJA treatment: 16 *CmTH* genes were induced at 24 h, whereas *CmTH2* and *CmTH9* were induced at 4 h but later repressed; conversely, *CmTH19* and *CmTH20* were not significantly altered (Figure 4b). As an antagonist of MeJA, SA repressed the expression of the most of the *CmTH* genes, except for *CmTH12* and *CmTH15*/*19*, who were induced at 1 and 4 h, respectively (Figure 4c).

### 2.6. Expression Profiling of CmTH Genes under Abiotic Stress

**Figure 5:**
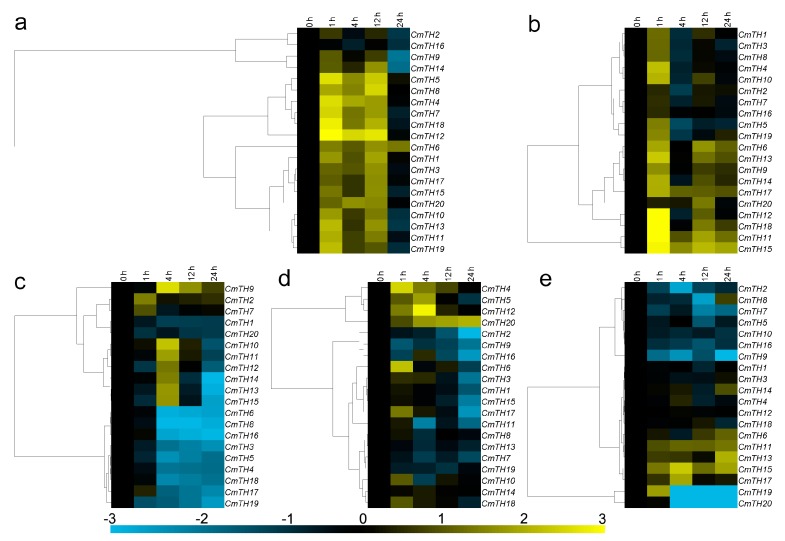
Differential transcription of *CmTH* genes in leaves as induced by (**a**) salinity stress; (**b**) drought stress; (**c**) low temperature (4 °C); (**d**) high temperature (40 °C); and (**e**) wound treatments. Blue and yellow indicate lower and higher transcript abundance, respectively, compared to the relevant controls. Bar at the bottom represents log_2_ transformed values.

Three main expression patterns of *CmTH*s were observed under salinity stress. Although the expression of *CmTH2* and *CmTH16* was not significantly altered by NaCl treatment, *CmTH9* and *CmTH14* were suppressed at 24 h; in contrast, the other sixteen *CmTHs* were up-regulated at 1, 4 and 12 h (Figure 5a). Six *CmTH*s (*1*, *2*, *3*, *7*, *8*, and *16*) were weakly regulated by drought stress, with a range of variation less than two-fold. *CmTH11*, *CmTH12*, *CmTH15* and *CmTH18* were markedly induced at 1 h after PEG treatment, whereas eight *CmTHs* (*4*, *6*, *9*, *10*, *13*, *14*, *17*, and *20*) were only slightly induced. Furthermore, *CmTH5* and *CmTH19* were up-regulated by high osmotic pressure at 1 h but were repressed thereafter (Figure 5b). After exposure to low temperature, nine of the 20 *CmTH* genes (*3*, *4*, *5*, *6*, *8*, *16*, *17*, *18*, and *19*) were strongly suppressed at 4 h. The transcript abundance of *CmTH9* was increased at 4 h, and that of *CmTH10*, *CmTH11*, *CmTH12*, *CmTH13*, *CmTH14*, and *CmTH15* was also increased at 4 h but decreased thereafter; the other four *CmTH*s (*1*, *2*, *7*, and *20*) were weakly regulated by low temperature, with a range of variation less than two-fold (Figure 5c). Eight *CmTH* genes (*1*, *2*, *3*, *9*, *11*, *15*, *16*, and *17*) were down-regulated by high temperature, whereas five (*4*, *5*, *6*, *12*, and *20*) were up-regulated. The other seven *CmTH*s were not significantly affected by high temperature (Figure 5d). With the exception of *CmTH12*, the *CmTH* genes were all down-regulated by high temperature, with *CmTH4* and *CmTH5* transcript expression being decreased more than eight-fold with respect to that at 0 h (Figure 4g). Five *CmTHs* (*2*, *7*, *8*, *9*, and *20*) were significantly repressed by mechanical damage, whereas *CmTH19* was increased at 1 h but repressed after 4 h. *CmTH15* was up-regulated at 4 and 24 h; *CmTH13* and *CmTH17* were down-regulated at 24 and 4 h, respectively. The expression of the other 11 *CmTHs* was not significantly altered by mechanical damage (Figure 5e).

## 3. Discussion

### 3.1. The Trihelix Family in Chrysanthemum

In general, trihelix TFs are thought to feature a typical DNA-binding trihelix (helix-loop-helix-loop-helix) structure [21]. A few trihelix genes from various plants have recently been cloned and characterized, showing great functional divergence in processes such as seed scattering during crop domestication, embryo development, morphogenesis control of various flower organs, and resistance to biotic and abiotic stresses [22]. Most of these functions have been studied in *Arabidopsis*, whereas no members of the family have been isolated from flower crops, e.g., chrysanthemum. By taking advantage of the availability of the chrysanthemum transcriptome, we identified 20 members of this family on the basis of sequence homology using putative trihelix genes as blast queries. The trihelix family has been classified based on the genes in *Arabidopsis* into five distinct subfamilies: GT-1, GT-2, GTγ, SIP1, and SH4 [3]. In the present study, we compared *Arabidopsis* and chrysanthemum sequences and constructed a phylogenetic tree using the neighbor-joining procedure to classify the genes into five major clades (Figure 1). The results are consistent with those of Kaplan-Levy [3]. Our motif analysis also confirmed the classification, with each clade containing specific motifs (Figure 2). Three *Arabidopsis* genes and two from chrysanthemum (CmTH9 and CmTH10) are found in clade GTγ, though the function of *Arabidopsis* genes have not been reported in this clade previously. After searching the TAIR database, 13 out of 30 *Arabidopsis* trihelix family proteins were located in the nucleus, six proteins were located in the nucleus and other cellular components, nine proteins were located in other cellular components, and two proteins’ subcellular localization was unknown. Our result was consistent with it, assessment of the subcellular localization of chrysanthemum trihelix genes revealed strong support (twelve genes) for their functional roles in transcriptional regulation (Table 1). Nonetheless, the transcriptional activity of these family members needs to be investigated further.

### 3.2. Diverse Motifs Predicted in TH Factors

We further analyzed conserved motifs among chrysanthemum trihelix family members using the MEME program and found that the majority of the CmTHs in the same group shared similar motifs, suggesting that these conserved motifs play crucial roles in group-specific functions. However, high divergence in structure was found among the different groups. For example, the GT-1 and GT-2 subfamilies contain Motif 6, whereas the GT-2 and GTγ subfamilies contain Motif 7 (Figure 2), which reflects the complex nature of the function of TH proteins in chrysanthemum. We also found motifs conserved in certain subfamilies, e.g., Motif 3 in the SIP1 clade and Motif 10 in the GTγ clade. The distribution of motifs indicated that the genes containing the same motifs were likely produced through gene expansion within the same group.

Since they all form a helix-turn-helix structure, trihelix TFs are classified into the Myb/SANT-like family (PF13837) in Pfam. In our study, Motif 1 matched the Myb-type HTH (helix-turn-helix) DNA-binding domain (IPR017930). However, the trihelix family is generally thought to feature a typical helix-loop-helix-loop-helix structure with individual helices longer than the myb repeat, which targets different DNA sequences [23].

### 3.3. miRNA Target Site Prediction

To our knowledge, reports on miRNA-TH interactions are rare. Our predictions indicated that 16 *CmTH*s can be targeted by 20 miRNA families, but only three *Arabidopsis* GT-2 subfamily genes can be targeted by 5 miRNA families (Table S1). Moreover, we predicted that miR414 can target nine *CmTH*s (distributed among three subfamilies, GT-2, GTγ, and SIP1), which suggests that this interaction module is conserved in the chrysanthemum trihelix family. Regardless, these interaction regulation pathways in plants should be verified by further research.

### 3.4. Organ-Preferential Expression of CmTH Genes

Since gene expression patterns can provide important clues for gene function, we employed qRT-PCR to examine the expression of *CmTH* genes in the roots, stems and leaves of young plants as well as in the tube and ray florets of inflorescences at the bud stage and in pollen (Figure 3). The expression profiles obtained reveal spatial variations of *CmTH* expression in different organs. Furthermore, five pairs of paralogous genes (except *CmTH13* and *CmTH14*) showed distinct expression patterns, suggesting that significant functional divergence might have occurred following duplication events.

As shown in Figure 3, most chrysanthemum trihelix genes were highly expressed in inflorescences, which is consistent with previously described functional roles of trihelix genes during flower development [3]. *CmTH1*, *CmTH7*, and *CmTH18* showed relatively high expression levels in stems, whereas *CmTH17* and *CmTH20* were highly expressed in leaves, suggesting that they might play a role in the development of the stem or leaf, respectively. However, additional research is needed to determine the functions of these *CmTH* genes.

### 3.5. Transcriptional Responses of Chrysanthemum Trihelix Genes after Phytohormone or against Abiotic Stress Treatment

As plant trihelix family TFs play very important roles in development and stress response [24], we investigated the responses of *CmTH*s to different plant hormones and abiotic stresses. *CmTH*s were both up-regulated and down-regulated by the treatments (Figure 4 and Figure 5), indicating that *CmTH*s may be involved in responses to various plant hormones that elicit a stress response.

In *Arabidopsis*, *GT-1* and related *GT-4* are expressed ubiquitously [25], and their homolog in chrysanthemum, *CmTH7*, is also constitutively expressed in various organs. *Arabidopsis* *GT-2-LIKE1* function was revealed to limit the extent of endoreduplication by modulating the expression of relevant cell cycle genes [26], and the expression of *GT-2-LIKE1* is reduced during drought stress, possibly leading to the generation of fewer stomata in newly arising leaves [27]. On the other hand, soybean *GmGT-2B* is most closely related to *GT-2-LIKE1*, its expression is induced by ABA, high salt, drought, and cold in 15-day-old seedlings [11]. In chrysanthemum, its ortholog, *CmTH20*, was found to be induced by ABA, NaCl, PEG, and high-temperature treatments, suggesting that it may have functions similar to soybean *GmGT-2B*, as opposed to *Arabidopsis GT-2-LIKE1*.

The functions of the GTγ clade have only recently been investigated. Expression of *OsGT*γ*-1*, *OsGT*γ*-2*, and *OsGT*γ*-3* in rice seedlings can be induced by salt, drought, cold, and ABA treatments [28]. There are three GTγ group genes in *Arabidopsis*, though expression of these genes does not appear to exhibit similar stress-induced trends [29]. Our expression data showed induction of GTγ-clade genes in chrysanthemum (*CmTH9* and *CmTH10*) by salt, drought, cold, and JA treatments, and further studies on the extent of such stress-related functions will be of interest. The first member of the SIP1 clade, NtSIP1, was identified in tobacco [30], and two SIP1 group genes (*ASIL1* and *ASIL2*) from *Arabidopsis* have recently been shown to repress the expression of late embryo development genes in seedlings [31]. However, their responses to abiotic and biotic stresses have not been reported to date. Thirteen of twenty chrysanthemum trihelix genes belong to the SIP1 clade, and their transcriptional responses under phytohormone or abiotic stress treatments were analyzed. For instance, most of *CmTH*s in SIP1 clade (except *CmTH2* and *CmTH14*) were induced by NaCl stress, furthermore, *CmTH11*, -*12*, -*15* and -*18* were markedly induced by PEG treatment. It strongly suggested that SIP1 clade *CmTH* genes were participated in the abiotic stresses response of chrysanthemum. Our results will promote future research on the biological functions of genes in this clade.

## 4. Materials and Methods

### 4.1. Plant Materials and Growth Conditions

Cuttings of the cut-flower chrysanthemum cultivar “Jinba”, maintained at the Chrysanthemum Germplasm Resource Preservation Center (Nanjing Agricultural University, Nanjing, China), were rooted in vermiculite without fertilizer in a greenhouse. After 14 days, the plants were transplanted to a growth substrate and subjected to a range of stress and phytohormone treatments.

### 4.2. Database Searches and Sequencing of Full-Length CmTH cDNAs

All of the putative trihelix proteins were retrieved from *C. morifolium* transcriptome data, which was derived from petal and leaf tissues [32]. *Arabidopsis* trihelix protein sequences were downloaded from The Arabidopsis Information Resource (TAIR) database and used as query sequences to identify CmTH proteins. Multiple alignments among the identified CmTH sequences were also performed to avoid repetition. Furthermore, the full open reading frames of *CmTH*s were obtained via RACE PCR. First-strand cDNA was synthesized using the dT adaptor primer dT-AP and then subjected to nested PCR using the primer pair CmTHx-3-F1/F2 and the adaptor primer AP (Table S2). Finally, the complete open reading frames (ORF) of 20 *CmTH*s were amplified using Phusion^®^ High-Fidelity PCR kit with gene-specific primers (Table S3). The amplicons were purified using AxyPrep DNA Gel Extraction Kit (Axygen, Hangzhou, China) and cloned into pMD19-T (TaKaRa, Tokyo, Japan) for sequencing. The consistent sequence of each *CmTH* was used to subsequent analysis.

### 4.3. Phylogenetic Tree Construction and Sequence Analysis

A phylogenetic tree was constructed with MEGA version 6.0 using the neighbor-joining method [33]. ClustalW software was employed for multi-sequence alignments of trihelix TFs between *Arabidopsis* and *C. morifolium* [34]. Computation of the theoretical isoelectric point (pI) and molecular weight (*M*w) of CmTH proteins was performed using the Compute pI/*M*w online tool [35], and PSORT [36] was used to predict subcellular localization. Putative conserved motifs in these collected trihelix proteins were predicted using the MEME program v4.10.2 [37]; the number of motifs was set to 10. All motifs identified by MEME were searched in the InterPro database using Inter-ProScan [38]. Target prediction for miRNA was performed using the psRNATarget online tool [39].

### 4.4. Plant Treatments

Tissue-specific transcription profiles of 20 *CmTH* genes were explored in roots, stems, and leaves of young plant,s as well as in tube and ray florets of inflorescences at the bud stage and pollen.

A variety of abiotic stresses were imposed, including high salinity (200 mM NaCl) and drought (20% *w*/*v* polyethylene glycol (PEG6000)) [40]. For NaCl and PEG6000 assays, young plants were transferred to liquid medium containing the stress agent, and the second true leaves were sampled at various time points [41]. Other plants were subjected to a period of exposure at either 4 or 40 °C in a chamber under a 16 h photoperiod with 50 µmol·m^−2^·s^−1^ of light; the second true leaves were sampled [42]. A wounding treatment involved cutting the second true leaf, and phytohormone treatments involved spraying the leaves with either 50 µM ABA, 1 mM MeJA, or 200 µM SA [43]. For all treatments, the second true leaf of each plant was sampled with three biological replicates, prior to stress treatment and then at 1, 4, 12, and 24 h.

After sampling, all of the collected material was snap frozen in liquid nitrogen and stored at −70 °C. Each treatment was replicated three times.

### 4.5. Real-Time Quantitative PCR (qPCR)

Total RNA was isolated from samples using the RNAiso reagent (TaKaRa) according to the manufacturer’s instructions. The RNA was then treated with RNase-free DNase I (TaKaRa) to remove potential genomic DNA contamination. First-strand cDNA was synthesized from 1 µg of total RNA using SuperScript III reverse transcriptase (Invitrogen, Carlsbad, CA, USA) according to the manufacturer’s instructions. qPCR was performed using a Mastercycler EP Realplex instrument (Eppendorf, Hamburg, Germany). Each 20 µL amplification reaction contained 10 µL SYBR^®^ Premix Ex Taq™ II (TakaRa), 0.4 µL each primer (10 µM), 4.2 µL H_2_O and 5 µL cDNA template. The PCR cycling regime consisted of an initial denaturation (95 °C/2 min) followed by 40 cycles of 95 °C for 10 s, 55 °C for 15 s, and 72 °C for 20 s. A melting curve analysis was performed following each assay to confirm the specificity of the amplicons. Gene-specific primers (provided in Table S4) were designed using Primer3 Release 2.3.4 [44], and the *EF1α* gene was employed as a reference sequence [43]. Relative transcript abundance was calculated by the 2^−ΔΔ*C*t^ method [45]. For each biological replicates, three technical repeats were performed.

### 4.6. Data Analysis

The relative expression levels of each *CmTH* gene were log_2_ transformed. The profiles were compared using Cluster v3.0 software [46] and visualized using Treeview [47]. The expression data were analyzed by Student’s *t*-test using the SPSS v17.0 software (SPSS Inc., Chicago, IL, USA).

## 5. Conclusions

This study is the first transcriptome-wide analysis of the trihelix TF family in chrysanthemum. The phylogenetic analysis revealed twenty *CmTH* genes were distributed across four of five groups (except SH4), while transcriptional analysis displayed the expression of 20 *CmTHs* was in response to a range of phytohormones and abiotic stress treatments. Additionally, bioinformatics analysis predicted that 16 *CmTH*s can be targeted by 20 miRNA families and miR414 can target nine *CmTH*s. Our findings lay a foundation for future research on the biological functions of the members of this family, including localized growth suppression and responses to abiotic stresses. Future studies on *CmTH* genes will shed light on the fundamental functions of these genes and promote their application in chrysanthemum breeding.

## Supplementary Materials

Supplementary materials can be found at http://www.mdpi.com/1422-0067/17/2/198/s1.
