# Supplementary material for: Transcriptome-Wide Identification and Expression Profiling Analysis of Chrysanthemum Trihelix Transcription Factors
Source: Int J Mol Sci. 2016 Feb 2;17(2):198. doi: 10.3390/ijms17020198 (PMC4783932; doi:10.3390/ijms17020198)
Supplement: Supplementary file 1 [file ijms-17-00198-s001.zip › ijms-113359-Supplementary Materials/ijms-113359-Supplementary.pdf]

Supplementary Materials: Transcriptome-Wide Identification and Expression Profiling Analysis of Chrysanthemum Trihelix Transcription Factors

Aiping Song, Dan Wu, Qingqing Fan, Chang Tian, Sumei Chen, Zhiyong Guan, Jingjing Xin, Kunkun Zhao and Fadi Chen

	Motif	E-value	Sites	Width
1.	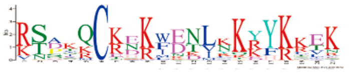	1.3×10^{-432}	50	21
2.	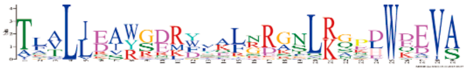	1.7×10^{-305}	29	29
3.	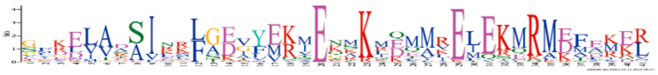	4.9×10^{-249}	22	41
4.	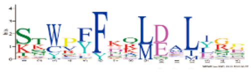	5.6×10^{-141}	37	15
5.	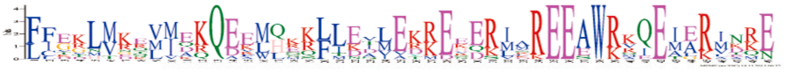	8.1×10^{-137}	11	49
6.	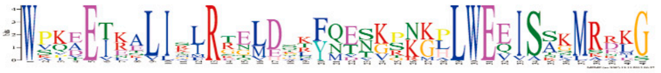	2.9×10^{-131}	11	41
7.	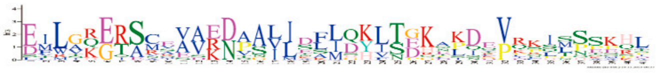	1.2×10^{-072}	13	41
8.	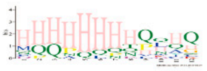	2.6×10^{-053}	14	12
9.	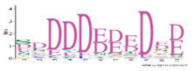	1.5×10^{-048}	37	11
10.	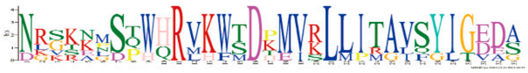	1.8×10^{-045}	6	33

Figure S1. Conserved amino acid sequences of motif identified by MEME in trihelix transcription factors.
